# Providing an optimized model to detect driver genes from heterogeneous cancer samples using restriction in subspace learning

**DOI:** 10.1038/s41598-021-88548-2

**Published:** 2021-04-28

**Authors:** Ali Reza Ebadi, Ali Soleimani, Abdulbaghi Ghaderzadeh

**Affiliations:** 1 Department Computer Engineering, Sanandaj Branch, Islamic Azad University, Sanandaj, Iran; 2 Department of Computer Engineering, College of Technical and Engineering, Malard Branch, Islamic Azad University, Tehran, Iran

**Keywords:** Cancer genomics, Cancer prevention, Cancer screening

## Abstract

Extracting the drivers from genes with mutation, and segregation of driver and passenger genes are known as the most controversial issues in cancer studies. According to the heterogeneity of cancer, it is not possible to identify indicators under a group of associated drivers, in order to identify a group of patients with diseases related to these subgroups. Therefore, the precise identification of the related driver genes using artificial intelligence techniques is still considered as a challenge for researchers. In this research, a new method has been developed using the subspace learning method, unsupervised learning, and with more constraints. Accordingly, it has been attempted to extract the driver genes with more precision and accurate results. The obtained results show that the proposed method is more to predict the driver genes and subgroups of driver genes which have the highest degree of overlap due to *p*-value with known driver genes in valid databases. Driver genes are the benchmark of MsigDB which have more overlap compared to them as selected driver genes. In this article, in addition to including the driver genes defined in previous work, introduce newer driver genes. The minister will define newer groups of driver genes compared to other methods the *p*-value of the proposed method was 9.21e-7 better than previous methods for 200 genes. Due to the overlap and newer driver genes and driver gene group and subgroups. The results show that the *p* value of the proposed method is about 2.7 times less than the driver sub method due to overlap, indicating that the proposed method can identify driver genes in cancerous tumors with greater accuracy and reliability.

## Introduction

Cancer is one of the deadliest diseases, and according to the estimation of the American Cancer Association in 2019, about 1,762,450 people has cancer worldwide, of them about 606, 880, individuals have died. Cancer is the second leading cause of death among all diseases^1^. One of the reasons for the abnormal tumor growth, is the rate of DNA mutation in the driver genes, which consequently causes mess in the function of the cancerous cell of a tumor. Due to this reason, having integrated information on this field helps establishing cancer detection and treatment strategies^2^. The large genome changes is one of the causes of cancer, using the second-generation technology of DNA sequencing and analysis, which would significantly contribute to the biological understanding of diagnosis and treatment of cancer. This insight helps us examining each type of change in the somatic genome, and also facilitates the detection of mutant genes in cancer samples^3^. In this study, although we have been able to identify all mutant genes in the tumor, many of these mutant genes have no effect on the tumor development, which are known as passenger genes. Accurate and direct identification of whether this passenger gene has an impact on the development of the tumor or not, still remains a challenge. So, one of the major works in the field of cancer research is identifying the passenger gene from the driver’s gene in cases with cancer^4,5^. One common way to deduce driver genes, is a hypothesis that “the driver's mutated genes are primarily among the large groups of sample mutated tumor genes”. Therefore, based on this hypothesis, many scientific studies have been driven using computational methods of identifying driver genes among the mutated gene groups in this field^6^. In OncodriveCLUST, specific genes that tend to cluster mutations throughout the protein sequence, were identified, which indicated that these genes have a particular bias toward their dependent gene sequences. Moreover, based on this hypothesis, in this method, a number of genes that had high mutation frequencies were the driving candidate genes, which were later found to have no significant effect on tumor growth^7^. The MutSigCV method solves one of the challenges in identifying driver genes. Previous methods have identified a list of driver genes, but because of mutation heterogeneity, some of them have not been identified properly. Therefore, by the use of this method, this problem has been solved^8^. Because cancer is a heterogeneous disease, there are many different subtypes for one type of cancer, and the driver genes of each subgroup may be different from the other genes. If a mutated gene acts as a driver gene for several specimens in a subgroup, it can be identified as the driver gene of the subgroup and also can be used as a criterion for separating subgroups^9^. Considering the genomic diversity and heterogeneity of subgroups of specific genes in a group, which their driver is small part of samples, so they are rarely changing among all the samples^10^. Other methods are also used to identify a rare mutation except for mutation frequency, such as modifying the amino acid of the flanking sequence Another method based on optimizing SpeMDP and the maximum matrix weight, is used to identify the driver genes. In this method, the genome data of twelve different types of heterogeneous cancer are used to form a common biological path, and finally the genes in this common path, are used as candidate genes^11,12^. All the results of the previous methods are encountered the problem that methods are suitable for the idea mode. It is appropriate when all subgroup information are available, which are not mostly available in many cases. The accurate extraction of the driver genes, without providing the subgroup information to find the exact treatment of cancer and personal medicine, still remains as a challenge^10^. To solve the problem of inaccessibility of information, the margin writing of the subgroups, as Driversub method was proposed. Correspondingly, in this method, an unsupervised learning method was used^13^, which needs no information about subgroup^14^. One of the challenges in analyzing the results of this method is that the available data has noise and there is still discarded data, which consequently affects the accuracy of the results. Therefore, in this study, we have tried to overcome this problem by developing this method. In this article, we achieved better accurate for this method via developing the driversub method, and by applying more restrictions on the data. To achieve this goal, robust adaptive graph regularized non-negative matrix factorization method has been used, and by applying less weight to noisy as well as the discarded data, and giving more weight to clean data, we have tried to improve the accuracy of the results. This article used the Cancer Genome Atlas (TCGA) program and Cancer Gene Gensus (CGC).

## Method

### Subspace learning

Due to the lack of subgroup information, we have used an unsupervised learning method. To do this, a subspace learning framework has been used^15^. Afterward, we displayed the marginal writing information of a gene, as a vector, so that the mutation data of the gene with high dimensions was converted into a small subspace with smaller dimensions. Gene mutation input data was converted to a binary matrix. The mutation vector of each gene is *X* = [*x*_1_, *x*_2_, *x*_3_,…*x*_*p*_], where p is equal to the total number of genes. The input matrix contains p-genes and n-samples, and each entry of this matrix indicates whether the ith gene has been mutated in the j sample or not^16^. The output matrix was *Z* = [z1, *z*_2_, *z*_3_,… *z*_*i*_ …*z*_*p*_], which was compressed space with less dimensions, so that k <  < n, where k is the dimension of the output matrix Z^17^. Low-dimensional output matrices in vector space can be better suited for the computational analysis. Although the output matrix can well represent the mutation index of the input matrix, the main challenge is that there is no indication to show that the investigated gene is from which one of the subgroups. In fact, there is no general criterion for matching a gene with a subgroup. Due to the fact that the sub-space dimensions can determine the hidden features related to each gene, and the sub-space output dimensions are almost able to determine the indicators related to each subgroup. However, there is no guarantee that the dimensions of the subspecies matrix represent those indicators related to that subgroup, so it can be used to determine whether the special checked genes is relevant to that subgroup^18^. Based on the two hypotheses proposed in the driversub method, the values of the output vector indices can be used as criteria for evaluating the driver's genes. Also, in the second hypothesis, the values of the output vectors can be used indicators for determining whether a gene belongs to a specific subgroup. However, to increase the guarantee of the first hypothesis in the driversub method, the regularization of L1 norm was used to ensure that the output vectors are sparse^19^. Because the values of the espresso of output vector index are large, the output vectors will more tend to be inclined to match the coordinates of the dimensions subspace. The axes of dimensions of subspace can be used as an indicator to recognize if a gene belongs to a particular subgroups. Hence, at the beginning of the first step, we use the objective function defined in the GNMF method ^20^as follows:

1 $$\mathop {\min }\limits_{U \ge 0,V \ge 0} \left\| {X - UV^{T} } \right\|_{F}^{2} + \lambda T_{r} (V^{T} L_{S} V)$$

where LS = DS -S is called graph Laplacian. S is the data similarity matrix, and DS is the degree matrix which the used attribute function in this method is as follows:

2 $$\begin{aligned} & \min \sum\limits_{\begin{subarray}{l} i = 1 \\ w,z \end{subarray} }^{p} {\mathop {\left\| {x_{i} - wz_{i} } \right\|}\nolimits_{2}^{2} } + \lambda_{z} \sum\limits_{i = 1}^{p} {\parallel z_{i} \parallel_{1} } \\ & {\rm{s}}{\rm{.t}} \;{\rm{W}} \ge 0\;\;{\rm{and}}\;\;Z_{i} \ge 0 ,\;\;\forall_{i} = 1, \ldots p \\ \end{aligned}$$

Which $\mathop \lambda \nolimits_{z}$ controls the distance between the output vectors Z and the coordinate axes and the coefficient of the regulator of sparse value. Also, one of the problems of the space learning method is an overflowing problem^21^. To overcome this problem, Frobenius norm regularization has been used in the driversub method, which changed the attribute function as follows.

3 $$\begin{aligned} & \mathop {\min }\limits_{{W,z_{i} }} \sum\limits_{i = 1}^{p} {\parallel x_{i} - Wz_{i} } \parallel_{2}^{2} + \lambda_{z} \sum\limits_{i = 1}^{p} {\parallel z_{i} } \parallel_{1} + \lambda_{w} \parallel W\parallel_{F}^{2} \\ & {\rm{s}}{\rm{.t}} \;W \ge 0\;\;{\rm{and}}\;\;Z_{i} \ge 0 .\;\;\forall_{i} = 1, \ldots ,p \\ \end{aligned}$$

Here, our parameters are the weight matrix W which can reverse the relationship between a subset of samples and subspace dimensions to calculate the real values of Matrix W and Z, we used the basic method of matrix factorization, and each time we repeated the initial W, Z we obtained more accurate values… What has been forgotten is that in calculating the similarity between the Samples, the Gaussian kernel function can also be used, which is as follows:

4 $$s_{i,j} = e^{{\frac{{\parallel x_{i} - x_{j} \parallel_{2}^{2} }}{{2\sigma^{2} }}}}$$

*s*_*i*,*j*_ is the similarity between i and j samples. While the Euclidean distance is used to compute the difference between two different samples, real trait space of sample including noise and large amounts of unrelated features, which play no role in similarity, but they can be used for similarity. To enhance the accuracy and precision, a number of irrelevant and disconnected attributes should be eliminated. It was shown that the attributes that have an impact, will have more weight in the distance calculation. Therefore, in order to achieve this goal, it is necessary to learn an M matrix to obtain the exact distance, so we have used M matrix in this article where M is a diagonal matrix. Herein, we get the distance as follows:

5 $$\parallel x_{i} - x_{j} \parallel_{M}^{2} = (x_{i} - x_{j} )^{T} M(x_{i} - x_{j} )$$

In this article, we have attempted to reduce noise by combining driversub. The methods as well as applying more restrictions on the obtained samples. Robust adaptive graph regularized NMF (RAGNMF) was also used, which is as follow:

6 $$\begin{aligned} & \mathop {\min }\limits_{w,z,W,M} Tr[M(x - wz_{i} )W(x - wz_{i} )^{T} ] + \lambda Tr(z^{T} l_{s} z) + \alpha \parallel W\parallel_{F}^{2} + \beta \parallel M\parallel_{F}^{2} \\ & {\rm{s}}{\rm{.t}} \;w \ge 0,\;\;z \ge 0,\;\;W \ge 0,\;\;M \ge 0 \\ \end{aligned}$$

## Optimization

To solve the desired method, a duplicate updating method was used, which is as follows.

By keeping W, M constant, the values of w and z were calculated as follows:

7 $$w_{ir} = w_{ir} \frac{{(MxWz)_{ir} }}{{(MwzWz^{t} )_{ir} }}$$

8 $$z_{jr} = z_{jr} \frac{{(Wx^{t} Mw)_{jr} }}{{(Wz^{t} w^{t} Mw + \lambda l_{s} z^{t} )_{jr} }}$$

In order to update the W value, by keeping values of M, w, and z constant, the following relationships were obtained.

9 $$\begin{aligned} & \mathop {\min }\limits_{w} = Tr[E^{M} W] + \alpha \parallel W\parallel_{F}^{2} \\ & {\rm{s}}{\rm{.t}}\;\;W_{i} \ge 0, \\ \end{aligned}$$

where *E*^*M*^ is as follows:

$$E^{M} = (x - wz)M(x - wz)^{T}$$

10 $$\sum\limits_{i = 1}^{n} {W_{i} } = C_{i}$$

Function (10) can be converted to the following equation:

11 $$\begin{aligned} & \min \sum\limits_{\begin{subarray}{l} i = 1 \\ M \end{subarray} }^{n} {\left( {W + \frac{{E_{i}^{M} }}{2\sigma }} \right)^{2}_{i} } \\ & {\rm{s}}{\rm{.t}}\;\;W_{i} \ge 0,\;\;\sum\limits_{i = 1}^{n} {W_{i} = C_{i} } \\ \end{aligned}$$

Also, by keeping the values of W, z, and w constant, the value of M was calculated as follows:

12 $$\begin{aligned} & \mathop {\min }\limits_{M} Tr[E^{W} M] + \beta \parallel M\parallel_{F}^{2} \\ & {\rm{s}}{\rm{.t}}\;\;M_{i} \ge 0,\sum\limits_{i = 1}^{m} {M_{i} = C_{i} } \\ \end{aligned}$$

We have used the matrix factorization method here Another point is that here $\lambda ,\alpha ,\beta$ are the control and regulating parameters and the correctness of the method depends on these parameters when the accuracy of the results is reduced when $\alpha$ is too large or too small, Here we have created a filter on the weights between the input and output vectors by imposing a constraint on the softness of the filter and low weights or high impact weights that are abnormal and the use of control parameters. We have achieved better results.

In this study, to solve this equation, we used the Accelerated Gradient Method, which was earlier used in^22^. The steps of performing this work are shown in the algorithm 1.1:
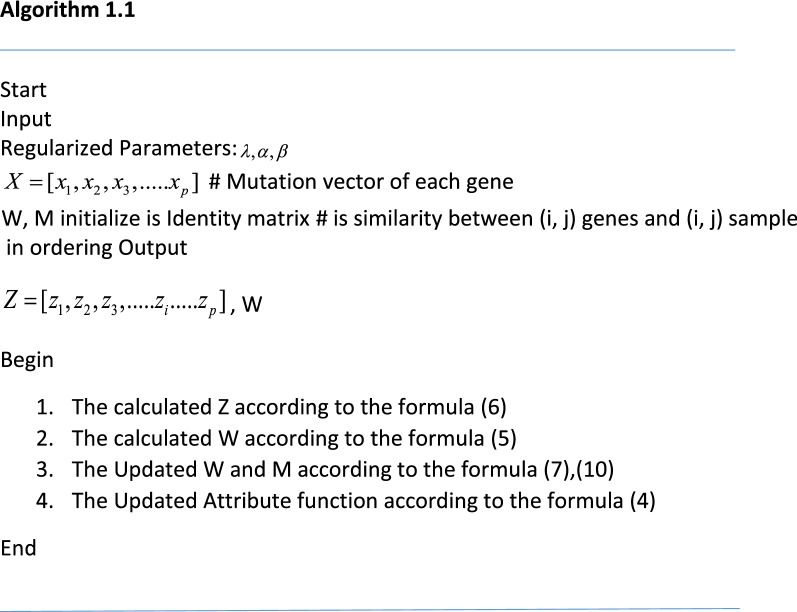


## Results

**Figure 1:**
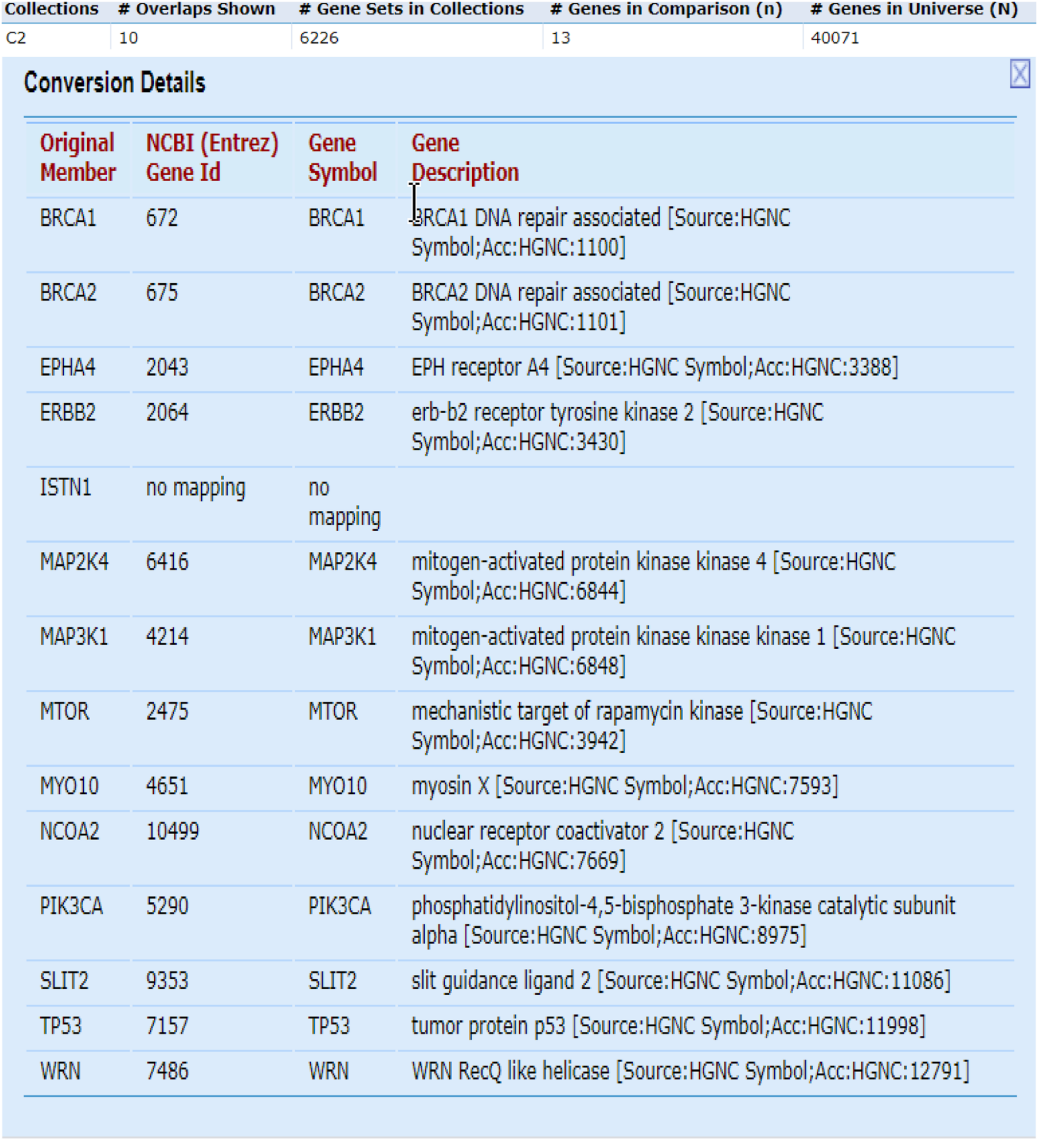
Details of 13 top driver genes in the proposed method.

**Figure 2:**
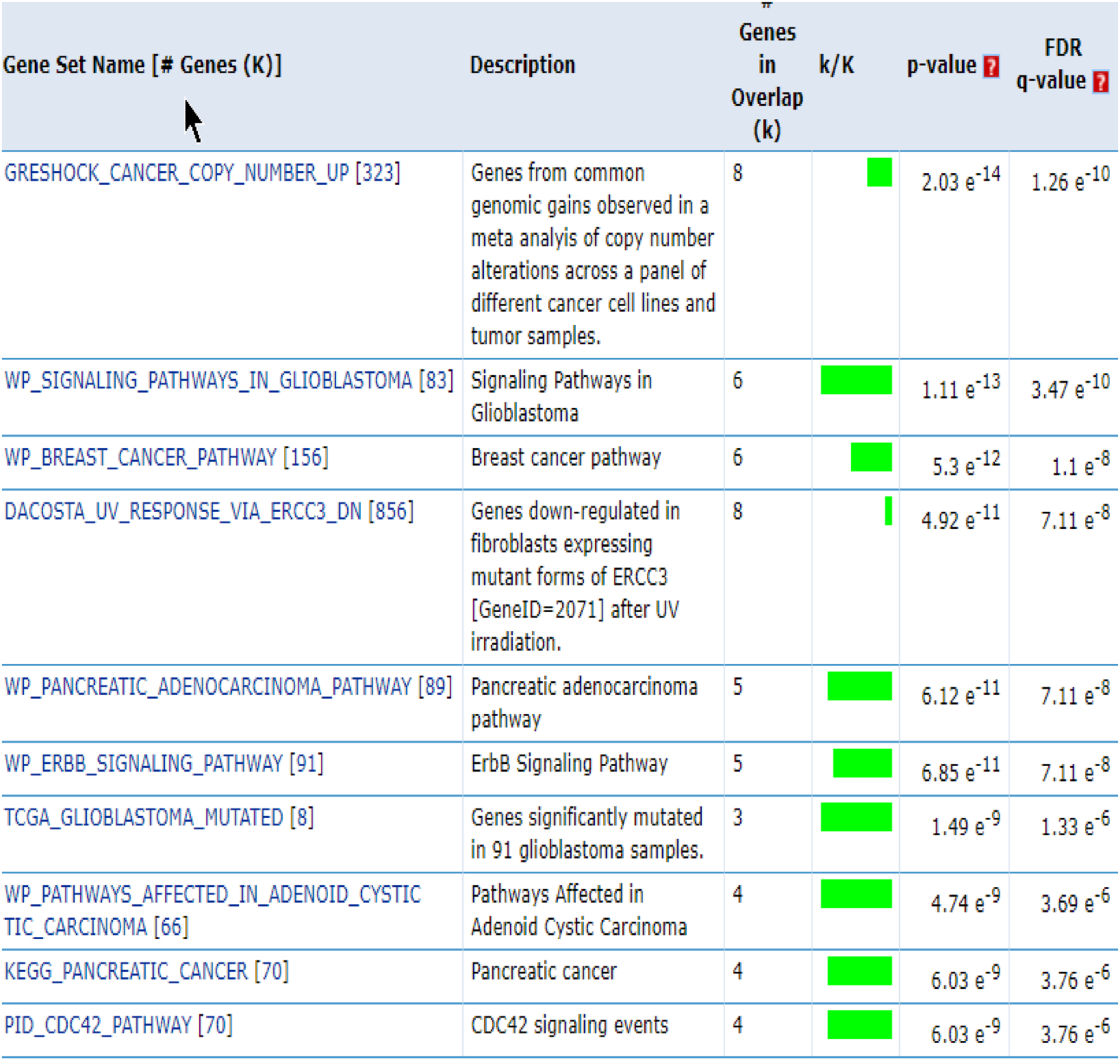
The number of driver genes overlapping with the benchmark dataset in a subset of the top 13 driver genes.

**Figure 3:**
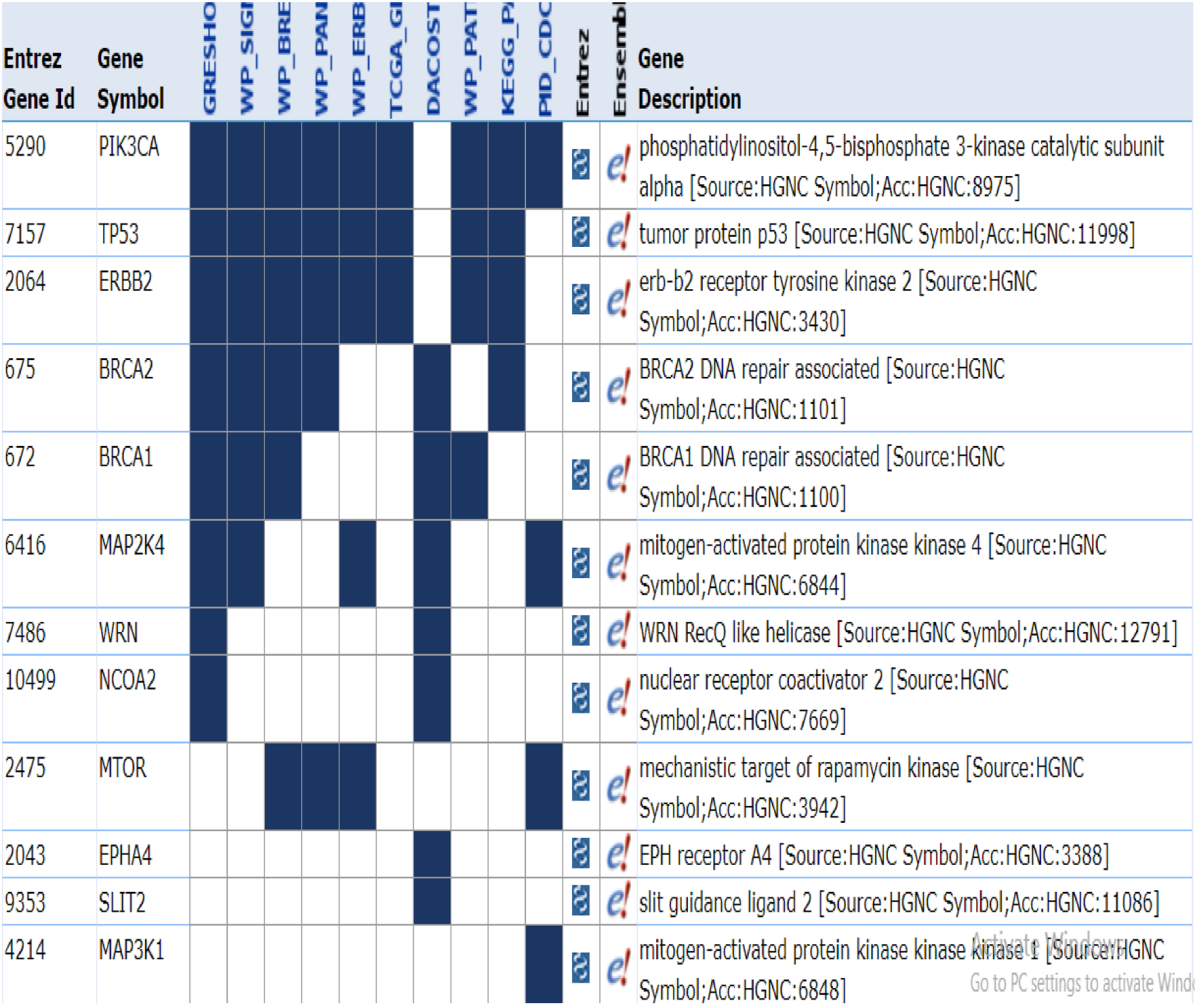
Proposed driver genes are compared to several benchmarks at the same time.

**Figure 4:**
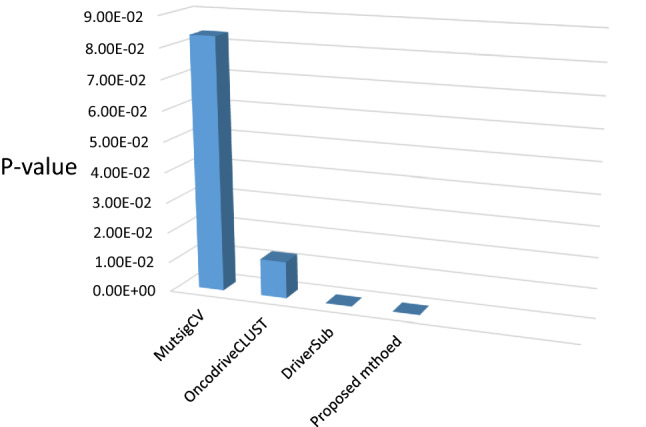
Comparing *p*-values between the previous and the proposed methods for an average of a subset of 200 driver genes.

**Figure 5:**
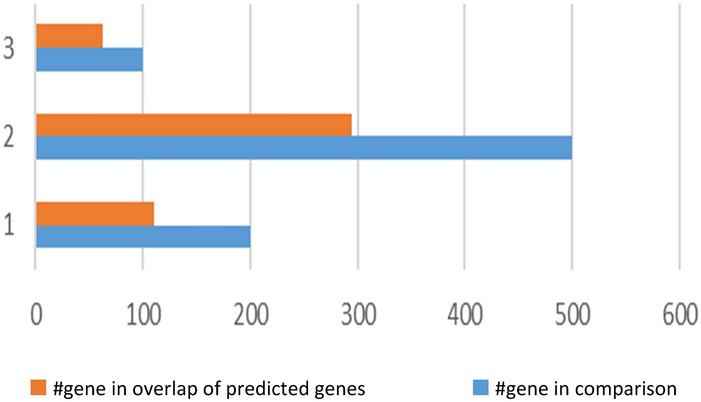
Overlap of the number of suggested genes with specific subgroups of 100, 200 and 500 genes of driver genes.

In this study, we used breast cancer data (Cancer Genome Atlas Network and others, 2012), which included somatic mutations of 507 samples and 12,233 genes that can be downloaded from the cBioPortal database^23^. By default, we considered the dimensions of k subspace as 4. In the present study, we have used Python 3.7 to implement this method. Moreover, we used Gsea Msigdb web-based software^24^ to analyze the results. Firstly, we calculated the mutation score of each gene from the output vector obtained from the learning subspace, and then arranged it in descending order. Thereafter, we separated the top 500 genes with the highest mutation scores, and then selected them as the candidate for driver genes. Finally, we compared the results with the Benchmarks on Msigdb show in Figs. 1, 2 and 3. Thus, we have taken from the 200 candidate driver genes obtained from this method about 13 genes which had the lowest *p*-value and highest mutation score, for example, and used the outputs obtained by the Msigdb web software and curated gene sets as a benchmark. Driver genes results have a very good overlap with the defined driver genes. They are also very similar to the previous methods in terms of defined driver genes. Besides, the new genes defined in this method are very similar due to *p*-value compared to previous works. They have better overlap with breast cancer benchmarks. The results of our simulation in the model presented in Table 1, Figs. 2 and 3. In Fig. 1, the details of candidate driver genes obtained from this method for breast cancer show 13 genes with characteristics which is based on the amount of *p*-value overlap with the driver genes in the specific subgroup of benchmark, As we can see in Fig. 2, from the 13 proposed driver genes in specific subgroups of the benchmark, we see the different numbers of genes of that overlap. Hence, the more driver genes overlap with the benchmark, and the lower the amount *p*-value, the better the outcome. In the Fig. 3, and as it can be seen, the more black cells there are, the more they overlap in one gene. In 200 driver genes obtained from top to bottom ranking, the number of genes proposed by our method overlapped better than previous methods so that we have achieved *p*-value = 9.21e-07. In Fig. 4 and Table 1, Comparing the proposed method with the previous methods due to the amount of *p*-value for 200 driver genes due to the averages can be seen, which the proposed method of this paper performs better. We compared the proposed specific subgroups of 100, 200, and 500 members of driver genes with the driver genes in the benchmark due to the degree of overlap. The results indicate a good degree of overlap. In details of Fig. 1, you can see 13 driver genes in the proposed method as showed in Table 1 and Fig. 4. Comparing *p*-values between the previous and the proposed methods for an average of a subset of 200 driver genes that the lowest *p*-value and highest mutation score which were compared by different methods in the Table 1. So that the proposed method has a significantly lower average *p*-value. Due to Fig. 5, the overlap of the number of suggested genes with curated gene sets (Misgdb) is observed. Considering the subgroups of 100, 200 and 500 members of driver genes are obtained and their overlap with the driver gene database is seen. The results indicate a good degree of overlap. to further analyze the results on breast cancer data, we compared the superior driver genes selected by existing method with previous methods so that 40 genes are shared between the proposed method and MutsigCV, and also between MutsigCV and Driversub. Under similar conditions, there are 21 common genes, and also between OncodriveCLUST and Driversub, there are about 81 common driver genes, while between the method proposed in this article and OncodriveCLUST, we had about 108 common genes which indicates that the proposed method is better. BRCA2, ERBB2, and PIK3CA are common, which are genes with high mutations, and also in the overlap between the proposed methods of OncodriveCLUST driver genes Which AKAP9, MTOR, TP53 are high mutant driver. The results indicate that the used method in this article with a high ability to predict and deduce driver genes was shown to be better than previous methods.

**Table 1:** Comparing *p*-values between the previous and the proposed methods for an average of a subset of 200 driver genes the results of comparison between different methods in the method proposed in this paper have an average of *p* value less for 200 driver genes.

Method	Average (*p* value)
MutSigCV	8.35e−02
OncodriveCLUST	1.23e−02
DriverSub	1.46e−06
proposed method	9.21e−07

Table 1 and Fig. 4 Comparing *p*-values between the previous and the proposed methods for an average of a subset of 200 driver genes the lowest *p* value and highest mutation score which were compared by different methods in the Table 1, so that the proposed method has a significantly lower average *p* value.

**Figure 6:**
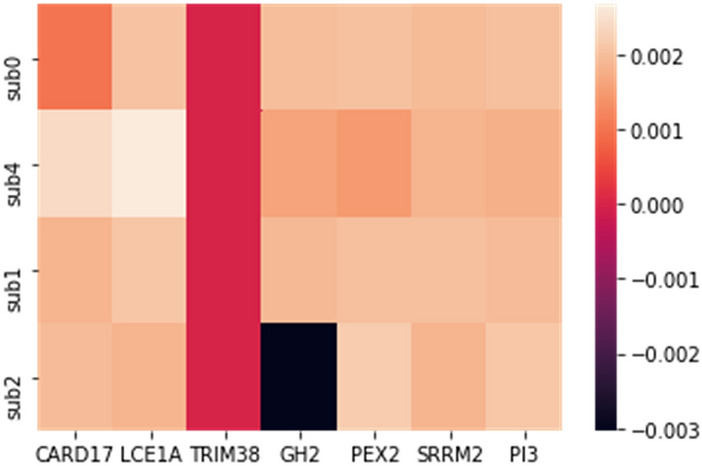
The amount of distribution *p* value of driver genes in the proposed method among specific subgroups.

In this method, BRCA1, BRCA2, ERBB2, PIK3CA, TP53, and KDM6A genes were introduced as driver candidate genes, which were also common in previous methods. The genes introduced by the proposed method, had a good overlap. In addition, the genes MYO10, ISTN1, EPHA4, SLIT2, WRN, DOP1B PLXNA2, and TCHH were introduced using the proposed method. Due to the elimination of overflow and suspension in the proposed method, the predicted genes were significantly different from the previous methods. Figure 6 shows the heat map diagram of seven genes with the highest score subspace (z) with k = 4 in the proposed method, which showed the heterogeneity of the mutation of specific genes in each one of the subgroups.

Figure 5 overlap of the number of suggested genes with curated gene sets (Misgdb) is observed. Considering the subgroups of 100, 200 and 500 members of the driver genes are obtained and their overlap with the driver gene database is seen. The results show that the candidate driver genes overlap well.

In Fig. 6, we see the overlap and distribution of the driver genes of new top candidate defined for 200 genes which were randomly selected with four specific subgroups of driver genes defined in the color bar, which the adjustment margin increases *p*-value from bottom to top. Black color indicates the lowest *p*-value and highest mutation score, for example, the gene GH2 has more overlap.

## Discussion

Extraction of subgroups of driver genes is one of the most important cases in personal medicine and heterogeneity in cancer. One of the problems in this regard is the lack of subspace margin information due to the fact that annotation of the subtypes of cancer samples is not available in many cases and previous methods cannot correctly determine the driver genes of each subgroup; hence, we predict the subtypes of driver genes in the heterogeneous cancers. A very important point which was forgotten in the past is that in calculating Z where our output is less than the input X under the confined space, the weight of input samples which are less important to influence the output of Z are not removed and cause the accuracy of Z matrix. In this work, we have achieved better results by creating constraints. In this method, we have used the subspace learning method and the unsupervised learning method. Due to the used method in this paper, more restrictions were applied on the distance between the input vector (X) and the output vector (z) in the subgroups, which was done by applying more weight to the samples that were more effective, and giving less weight to those that had no effect, and then applying it to the Euclidean distance between the two input and output vectors’ subspace. Herein, we attempted to extract the subgroups of the driver's genes more accurately. The results show that the proposed method can extract the driver genes more accurately and realistically compared to the previous methods. here There are many ideas for researchers to work with in the future, for example in^25,26^, the extraction of the characteristics of normal cancer cells through image processing using CNN and deep learning methods^27,28^ to isolate healthy cells from cancer, which can be done to identify the driver genes. Due to the openness of article subject, the researchers can achieve more accurate predictions from other methods such as deep learning and combining it with the method in this article. However, using CNN networks with computational complexity and high memory consumption due to the number FLOPS could be due to the volume of input data, and in this case, it should be improved by speeding up CNN through pruning methods. In fact, one of the advantages of using the method used in this article is the low computational complexity and low memory consumption compared to CNN which suffer from memory and computational complexity. Furthermore, in my future work, I decide to use deep learning and convolutional neural network (CNN) with the addition of other information genes in^29,30^ such as KEGG pathway and gene transcriptionally changes to more accurately predict specific subgroups of driver genes, Furthermore, using Weighted Gene Co-expression Network Analysis methods in^31^ for using in the body of the method of this paper to calculate the weight between input vector x and output vector z achieved better results. In future work, better results can be obtained for more accurate extraction to further analyze the results on breast cancer data.
